# Temporal Stability of Neural Markers of Motivated and Voluntary Attention: Case‐by‐Case and Group Analyses

**DOI:** 10.1111/psyp.70218

**Published:** 2025-12-31

**Authors:** Harald. T. Schupp, Karl‐Philipp Flösch, Ursula Kirmse, Tobias Flaisch

**Affiliations:** ^1^ Department of Psychology University of Konstanz Konstanz Germany; ^2^ Centre for the Advanced Study of Collective Behaviour University of Konstanz Konstanz Germany

## Abstract

There is growing evidence that electrophysiological markers of selective attention can be reliably assessed at the level of the individual case. The present study extends this work by examining their temporal stability across a one‐week retest interval, with a focus on motivated and voluntary attention. Seventeen healthy young adults were tested twice, 1 week apart, using a dense sensor EEG setup. Each session included blocks featuring either highly arousing erotic or mutilation images alongside low‐arousing control stimuli. To simultaneously assess voluntary attention, participants performed an emotion categorization task in which high or low arousal images served as targets in separate blocks. Across both emotion categories, the majority of participants showed significant emotional modulation of the EPN (88%) and LPP (100%) components, as well as target P3 effects (76%) in both sessions. Complementary classification approaches based on different significance thresholds further supported the consistency of these effects. Subsampling analyses revealed that although reducing trial numbers diminished reliability, robust effects, particularly for the EPN and LPP in response to erotic stimuli, remained detectable at moderate trial counts. Analyses at the group level revealed excellent test–retest reliabilities for the EPN (ICC > 0.91) and target P3 (ICC > 0.86). While reliability was good for the LPP (ICC > 0.60), the confidence intervals indicated substantial variability. Together, these findings demonstrate that neural correlates of both motivated and voluntary attention exhibit high test–retest reliability at the individual level, reinforcing their utility as biomarkers and highlighting the value of single‐case analyses in electrophysiological research.

## Impact Statement

The temporal stability of neural effects associated with motivated and voluntary attention represents a foundational component for consistent and reproducible empirical findings. In this study, we first demonstrate good‐to‐excellent test–retest stability of ERP components related to motivated (EPN, LPP) and voluntary (P3) attention at the group level. Additionally, a case‐by‐case analysis revealed these effects at the individual level, enriching group‐level interpretations by incorporating individual neural response patterns.

## 1 Introduction

Replicable experimental findings are essential for establishing empirical regularities in affective and cognitive neuroscience. Whereas group‐level research typically emphasizes consistency across studies, an alternative approach focuses on effects at the level of the individual. In this framework, each case is treated as a replication, enabling researchers to identify regularities that hold across individuals while also capturing meaningful variability (Danziger 1990; Lamiell 1998; Robinson 2011). Building on this case‐by‐case perspective, the present research examined the stability of neural biomarkers of selective attention over time. Specifically, we asked whether ERP effects can be consistently replicated in single cases with high statistical confidence, thereby demonstrating intra‐individual replication, or whether they fluctuate, reflecting variability that challenges their reliability.

The preferential processing of significant stimuli in the environment is an essential function of attention. One form of selective attention is related to the intrinsic significance of stimuli, referred to as motivated attention (Bradley and Lang 2007). Two ERP components are well‐established indices of motivated attention. Specifically, the early posterior negativity (EPN), occurring between 150 and 350 ms post‐stimulus onset, and the late positive potential (LPP), occurring between 350 and 700 ms post‐stimulus onset, exhibit larger amplitudes for emotionally arousing compared to low‐arousing control stimuli (Cuthbert et al. 2000; Junghöfer et al. 2001; Schupp et al. 2003). Recent studies have examined these effects at the level of the individual case. Across three stimulus domains (i.e., sexual reproduction, disease avoidance, and predator fear), the majority of participants showed significantly larger EPN and LPP components to high‐ compared with low‐arousing stimuli (Schupp et al. 2023; Schupp and Kirmse 2021, 2022), thus demonstrating conceptual replication (Sidman 1960).

Attention can also be voluntarily directed toward stimuli in the environment based on explicit task instructions. Numerous studies have explored this form of attention using tasks in which participants respond either to basic stimulus features, such as size, color, or shape, or to higher‐order stimulus categories, such as humans, animals, or transport vehicles (Codispoti et al. 2006; Delorme et al. 2004; Fabre‐Thorpe et al. 2001; VanRullen and Thorpe 2001). Consistently, target stimulus processing elicits a P3 wave, a positivity most pronounced over centro‐parietal regions, occurring between 300 and 700 ms post‐stimulus. The consistency of the target P3 effect at the individual level has been well‐documented in research on lie detection (Farwell and Donchin 1991; Rosenfeld 2020), and recent work has demonstrated that motivated and voluntary attention can be assessed concurrently within a single experimental paradigm (Schupp et al. 2023).

To date, no study has systematically examined test–retest reliability at the level of the individual case, despite its importance for identifying stable biomarkers and demonstrating intra‐individual replication of neural responses. If neural networks are stable, neural biomarkers of selective attention should likewise remain consistent across measurements. However, repeated testing can itself alter electrophysiological measures of selective attention through processes such as affective habituation, practice effects, or increased familiarity with the laboratory context. Demonstrating stability across measurements thus constitutes a direct *intra*‐subject replication of the experimental effect (Sidman 1960), a necessary prerequisite for distinguishing between stability and meaningful variability; that is, plasticity in response to learning experiences.

Previous research assessed the temporal stability of selective attention at the group level. For motivated attention, most studies have focused on the LPP component using emotional interrupt paradigms. Kujawa et al. (2013) found fair to good stability for parietal and occipital LPPs in 8‐ to 13‐year‐olds over a two‐year interval (ICCs = 0.55–0.73), while Pegg et al. (2019) reported poor to fair reliability for early LPP responses (0.39–0.50) across 6 years in 9‐ to 15‐year‐olds. Bondy et al. (2018) observed stronger correlations over a four‐week interval in young females (rs = 0.73–0.87), and McGhie et al. (2021) reported similar stability in adult females over 8 weeks (rs = 0.67–0.69). Across five sessions separated by 2–14 days, Weinberg et al. (2021) observed excellent LPP stability in adults aged 18–60 years for pleasant (ICC = 0.84) and unpleasant (ICC = 0.82) images, whereas stability was lower for neutral images (ICC = 0.54). In contrast, Macatee et al. (2021) found poor LPP stability (ICC = 0.25–0.37) in combat‐exposed veterans over 12 weeks. For voluntary attention, the P3 component has shown generally good stability in visual oddball tasks. Cassidy et al. (2012) reported an ICC of 0.80 for mean P3 amplitude over 4 weeks, and Morand‐Beaulieu et al. (2022) found good to excellent stability for the P3b peak across two oddball task variants (ICCs = 0.80 and 0.74) over 4 months. Notably, while P3 difference scores (target vs. non‐target) show better stability (ICCs = 0.63–0.73), emotional difference scores (e.g., pleasant–neutral) have often yielded only poor‐to‐fair reliability (Bondy et al. 2018; Macatee et al. 2021; McGhie et al. 2021).

The present study investigated the temporal stability of the EPN, LPP, and P3 components at the individual level across a one‐week test–retest interval. To assess emotional stimulus significance, participants viewed high‐ and low‐arousal images related to sexual reproduction and disease avoidance in separate blocks. To examine explicit stimulus significance, they performed a categorization task in which they pressed a button whenever an exemplar from the target category was presented. We hypothesized that the majority of participants would show consistent and statistically significant effects across both sessions, reflecting robust neural responses to emotionally salient and goal‐relevant stimuli. To strengthen interpretive confidence, we combined conceptual replication across conditions with direct intra‐individual replication across sessions and applied graded inferential criteria, that is, different *p*‐value thresholds. While group‐level metrics were included for context, our primary aim was to demonstrate that stable empirical regularities emerge at the single‐case level and persist over time. Establishing such temporal stability is a critical step toward validating these ERP components as dependable neural biomarkers of emotional and voluntary attention.

## 2 Materials and Methods

### 2.1 Participants

Eighteen healthy volunteers were recruited from the University of Konstanz campus. Data from one participant were excluded due to a technical issue during session 2. The final sample consisted of 17 participants (9 males, 8 females) with a mean age of 23.18 years (SD = 2.71, range: 20–32 years). All participants had normal or corrected‐to‐normal vision and were in good health at the time of testing. None reported a history of neurological or psychiatric disorders. Participants received either monetary compensation or course credit for their participation.

Sample size was determined with respect to the primary focus on case‐by‐case analysis, with the first case serving to establish the effect and the remaining 16 cases providing individual‐level replication. For the ICC analyses, a post hoc power calculation following Rathbone et al. (2015) with ICC_0_ = 0, α = 0.05 (two‐tailed), and *N* = 17 showed that the study had 39.5% power to detect an ICC of 0.40, 79.1% power for an ICC of 0.60, and 97.3% power for an ICC of 0.75.

The ethical committee of the University of Konstanz approved the experimental procedure, which adhered to the regulations of the Declaration of Helsinki. All methods were conducted in full compliance with the approved guidelines. Informed consent was obtained from all participants, who were debriefed after completing the experiment.

### 2.2 Stimuli

Stimulus materials were selected based on the behavior systems of sexual reproduction and disease avoidance (cf. Schupp and Kirmse 2022). For each system, stimuli comprised 10 images per category, either high or low in emotional arousal.

For the sexual reproduction system (Domjan 1994), high‐arousal images depicted couples in explicit erotic postures, while low‐arousal control images showed couples in romantic poses, such as hugging or kissing. For the disease avoidance system (Marks 1988; Neuberg et al. 2011), high‐arousal stimuli depicted bleeding, injured, or deformed human bodies (mutilation and injury), whereas low‐arousal control images featured uninjured humans in neutral poses. Images were selected from the International Affective Picture System (IAPS; Lang et al. 2005)^1^ and public domain sources, ensuring similarity in overall composition between high‐ and low‐arousal stimuli.

To minimize physical differences between categories, images were standardized for brightness and contrast across the red, green, and blue channels. Additionally, each image was presented in both its original and horizontally flipped orientation to control for effects of lateralization, resulting in 40 pictures per behavior system (20 high‐arousal and 20 low‐arousal).

To confirm a priori selection criteria for the experimental stimulus set, participants rated the stimulus materials for valence and arousal after each experimental block in both sessions (Bradley and Lang 1994). Data were analyzed using two‐way repeated‐measures ANOVAs with Picture Category (high vs. low arousal) and Session (Session 1 vs. 2) as factors.

Regarding valence ratings, erotic stimuli (M = 5.85, CI_95_ [5.30; 6.39]) did not differ significantly from neutral stimuli (M = 6.25, CI_95_ [5.86; 6.64]; Picture Category: *F*(1, 16) = 1.26, *p* = 0.279). In contrast, mutilation pictures (M = 2.57, CI_95_ [1.97; 3.18]) received significantly lower valence ratings than neutral pictures (M = 5.72, CI_95_ [5.42; 6.03]; Picture Category: *F*(1, 16) = 75.53, *p* < 0.001, $\eta_{p}^{2}$ = 0.825). Neither the main effect of Session nor the interaction with Picture Category was significant.

Analysis of arousal ratings confirmed that erotic pictures (M = 5.51, CI_95_ [4.86; 6.16]) were rated as more arousing than neutral images (M = 2.77, CI_95_ [2.13; 3.41]; Picture Category: *F*(1, 16) = 51.37, *p* < 0.001, $\eta_{p}^{2}$ = 0.763). Similarly, mutilation pictures (M = 6.44, CI_95_ [5.62; 7.25]) were rated as more arousing than neutral images (M = 2.68, CI_95_ [2.13; 3.23]; Picture Category: *F*(1, 16) = 81.00, *p* < 0.001, $\eta_{p}^{2}$ = 0.835). Neither the main effect of Session nor the interaction with Picture Category was significant.

### 2.3 Experimental Design

The study consisted of two sessions conducted on separate days, spaced 1 week apart. To control for variations in circadian rhythm, testing sessions were scheduled at the same time on both days. Each session consisted of two experimental blocks, presenting high‐ and low‐arousing pictures either from the sexual reproduction or disease avoidance systems. The order of these behavior systems was counterbalanced across participants and gender. For each participant, the same picture sequence was shown in both sessions.

Within each block, stimuli were presented 30 times each in a pseudo‐randomized order for each participant. This resulted in a total of 1200 trials, with 600 presentations for each arousal category (high vs. low) per behavior system. No more than three consecutive presentations of high‐ or low‐arousing pictures were allowed, and transition frequencies between picture categories were controlled. Picture sequences were the same in both sessions. Breaks were provided within (after 600 trials) and between blocks to allow participants to adjust their posture and rest.

Participants were instructed to press a button as quickly and accurately as possible whenever a target picture appeared. The target category was indicated to participants through on‐screen instructions and alternated every 200 trials, resulting in 300 target trials and 300 non‐target trials both for the high‐ and low‐arousal categories, respectively. Before each experimental block, participants were familiarized with the pictures from the two stimulus categories.

Pictures were displayed for 117 ms, preceded by a fixation cross shown for 117 ms. The intertrial interval (ITI) varied pseudo‐randomly between 733 and 1083 ms (M = 907 ms). Participants could respond until the end of the ITI. When making an error, a red X was shown for 150 ms on a black background followed by a black screen for 217 ms before the start of the next trial. EEG data recording lasted approximately 120 min including breaks.

Task performance was high with an average accuracy of 97%. However, neutral images were categorized with higher accuracy, which was the case for the erotic picture set (M_Erotic_ = 96.33%, CI_95_ [95.05; 97.61]; M_Neutral_ = 96.98%, CI_95_ [95.66; 98.30]; Picture Category: *F*(1, 16) = 5.16, *p* = 0.037, $\eta_{p}^{2}$ = 0.244) as well as the mutilation picture set (M_Mutilation_ = 96.34%, CI_95_ [94.35; 98.34]; M_Neutral_ = 97.08%, CI_95_ [95.57; 98.60]; Picture Category: *F*(1, 16) = 4.52, *p* = 0.049, $\eta_{p}^{2}$ = 0.220). No other effect reached significance.

Regarding reaction time, faster responses were observed for erotic (M = 399 ms, CI_95_ [377; 422]) compared to neutral stimuli (M = 427 ms, CI_95_ [401; 453]); Picture Category (*F*(1, 16) = 24.11, *p* < 0.001, $\eta_{p}^{2}$ = 0.601). For responses to mutilations, there were significant main effects of Picture Category (*F*(1, 16) = 10.34, *p* = 0.005, $\eta_{p}^{2}$ = 0.393) and Session (*F*(1, 16) = 6.29, *p* = 0.023, $\eta_{p}^{2}$ = 0.282), which were qualified by the interaction of Picture Category × Session (*F*(1, 16) = 5.05, *p* = 0.039, $\eta_{p}^{2}$ = 0.240). Accordingly, responses were significantly faster to mutilation than to neutral stimuli in session 1 (M_Mutilation_ = 392 ms, CI_95_ [372; 413], M_Neutral_ = 413 ms, CI_95_ [390; 435], *t*(16) = −4.40, *p* < 0.001, Cohen's *d* = −1.07), but not in session 2 (M_Mutilation_ = 408 ms, CI_95_ [386; 430], M_Neutral_ = 418 ms, CI_95_ [390; 445], *t*(16) = −1.73, *p* = 0.104).

### 2.4 EEG Data Acquisition and Preprocessing

Brain and ocular scalp potentials were measured with 256‐lead HydroCel Geodesic Sensor Nets comprising silver chloride‐plated electrodes (*HCGSN, Electrical Geodesics Inc., Eugene, OR*). Electrode impedances were kept below 50 kΩ, as recommended for this type of electroencephalogram (EEG) amplifier by the manufacturer's guidelines. Data were recorded continuously using a Net Amps 300 amplifier and Net Station 5.4 acquisition software (Electrical Geodesics Inc., Eugene, OR) with the vertex sensor as reference electrode. After applying a low‐pass filter at 100 Hz, satisfying the Nyquist criterion, EEG data were sampled at 250 Hz and digitized at 32‐bit resolution. EMEGS software (Peyk et al. 2011) was used for subsequent offline analysis. Data were offline filtered using a digital low‐pass filter with a half‐power cut‐off at 40 Hz (Butterworth IIR filter, order 19, stopband: −45 dB at 50 Hz) and a digital high‐pass filter with a half‐power cut‐off at 0.06 Hz (Butterworth IIR filter, order 4, stopband: −18 dB at 0.05 Hz). The data were subsequently corrected for ocular artifacts using an automated regression method (Schlögl et al. 2007), converted to average reference, and baseline‐adjusted to the 100 ms pre‐stimulus interval. Artifact rejection was performed based on an elaborate method for statistical control of artifacts specifically tailored for the analysis of dense sensor EEG recordings (Junghöfer et al. 2000). Trials with errors in the categorization task were excluded from analyses. On average, M = 504.7 (SD = 34.7) trials per participant per condition were available for analysis in the erotic block, corresponding to on average 84.1% (SD = 5.8%) of all trials. In the mutilation block, on average M = 502.5 (SD = 34.5) trials were retained, corresponding to 83.7% (SD = 5.8%) of all trials. Trial counts did not significantly differ across experimental cells for each analysis, all χ^2^(3) < 1.7, *p* > 0.65.

#### 2.4.1 EEG Signal Quality

The quality of EEG signals was assessed using a combination of visual inspection and quantitative metrics. Visually, the ERP waveform for each condition and sensor cluster of interest (described below) was compared against the “(±) reference” ERP. This reference was created by alternating the polarity of every second trial before averaging, effectively canceling out the event‐related signal to reveal the noise level superimposed on the standard ERP (Schimmel 1967). Quantitatively, signal quality was assessed by computing the 90% confidence interval of the signal‐to‐noise ratio (SNR), as outlined in Parks et al. (2016), for each individually analyzed case. A bootstrap procedure was employed to resample the signal‐to‐noise ratio for each case, defined as the logarithmic ratio (in decibels) of root mean square (RMS) activity in the sensor cluster of interest during the post‐stimulus period (1–750 ms) relative to the pre‐stimulus baseline (−100 to 0 ms). For each case, these calculations were based on the same number of trials as used in the main bootstrap analysis. A minimum threshold of 3 dB for the lower bound of the 90% SNR confidence interval was used to define acceptable signal quality (cf. Parks et al. 2016).

The minimum signal quality threshold of 3 dB was met in each individual case. Moreover, the lower bound of the SNR confidence interval showed comparable variability across experimental conditions. For the block presenting erotic images, the lower bound of the SNR 90%‐CI for the EPN cluster ranged in Session 1 from 11.95 to 25.15 dB (M_S1_ = 18.58, SD_S1_ = 3.50) for individual cases. In Session 2 it ranged from 13.99 to 26.37 dB (M_S2_ = 19.34, SD_S2_ = 3.71). For the LPP cluster, values ranged in Session 1 from 13.02 to 23.23 dB (M_S1_ = 17.20, SD_S1_ = 2.92) and in Session 2 from 10.32 to 21.87 dB (M_S2_ = 16.93, SD_S2_ = 3.67).

For the block presenting mutilation images, the lower bound of the SNR 90%‐CI for individual cases ranged for the EPN cluster in Session 1 from 11.98 to 25.10 dB (M_S1_ = 19.17, SD_session1_ = 3.41) and in Session 2 from 13.72 to 26.76 dB (M_S2_ = 19.63, SD_S2_ = 3.42). For the LPP cluster, values ranged in Session 1 from 10.42 to 24.46 dB (M_S1_ = 16.78, SD_S1_ = 3.79) and in Session 2 from 9.51 to 22.89 dB (M_S2_ = 16.90, SD_S2_ = 3.53).

For the target P3 cluster, values in the block presenting erotic images ranged in Session 1 from 13.68 to 25.94 dB (M_S1_ = 16.30, SD_S1_ = 2.59) and in Session 2 from 10.43 to 22.89 dB (M_S2_ = 16.20, SD_S2_ = 3.61). In the block presenting mutilation images, values ranged in Session 1 from 12.14 to 24.12 dB (M_S1_ = 15.34, SD_S1_ = 2.99) and in Session 2 ranged from 12.73 to 20.21 dB (M_S2_ = 16.25, SD_S2_ = 2.61).

### 2.5 Group Analysis

#### 2.5.1 Sensor Clusters

EPN and LPP effects were pronounced in the group analyses, and single sensor waveform analyses were conducted to identify sensor clusters used for single subject bootstrap analyses. To assess emotional modulation of the EPN and LPP components, data were merged across the two sessions, and single‐sensor t‐tests were conducted separately for erotic and mutilation blocks. EPN and LPP sensors were selected showing significant effects at the level of *p* < 0.01 across all time points and both analyses in the EPN (200–260 ms) and LPP time window (380–480 ms).

The EPN was scored as mean activity in an occipito‐parietal sensor cluster comprising the following sensors: 83, 84, 85, 91, 92, 93, 94, 95, 96, 97, 102, 103, 104, 105, 106, 107, 108, 111, 112, 113, 114, 115, 116, 117, 120, 121, 122, 123, 124, 125, 126, 133, 134, 135, 136, 137, 138, 139, 145, 146, 147, 148, 149, 150, 151, 156, 157, 158, 159, 160, 161, 165, 166, 167, 168, 169, 170, 171, 174, 175, 176, 177, 178, 179, 187, 188, 189, 190, 191, 199, 200, 201, 208, 209, and 216.

The LPP was scored in a central cluster including the following sensors: 7, 8, 9, 16, 17, 24, 30, 41, 42, 43, 44, 45, 50, 51, 52, 53, 57, 58, 59, 60, 64, 65, 66, 71, 72, 76, 77, 78, 79, 80, 81, 87, 88, 89, 90, 100, 101, 129, 130, 131, 132, 142, 143, 144, 153, 154, 155, 163, 164, 172, 173, 181, 182, 183, 184, 185, 186, 194, 195, 196, 197, 198, 204, 205, 206, 207, 214, 215, and 257.

To analyze emotional modulation effects, separate repeated measures ANOVAs were then calculated for the erotic and the mutilation conditions with the factors of Picture Category (high vs. low arousal) and Session (1 vs. 2).

The same procedure was used to assess the target P3 effect. Merging data across sessions, single‐sensor t‐tests contrasting target and non‐target conditions were conducted for the erotic and mutilation conditions separately, and the sensor cluster selected on the p‐criterion of *p* < 0.01 in both analyses. The P3 was scored as mean activity in a time window from 420 to 520 ms in a parietal sensor cluster comprising the following sensors: 45, 53, 59, 60, 66, 77, 78, 79, 80, 81, 86, 87, 88, 89, 90, 98, 99, 100, 101, 109, 110, 119, 128, 129, 130, 131, 132, 140, 141, 142, 143, 144, 152, 153, 154, 155, 162, 163, 164, 183, and 257. To assess target effects, separate repeated measures ANOVAs were calculated for erotic and mutilation conditions with the factors of Target (target vs. non‐target) and Session (1 vs. 2).

Assumption checks using the Cramér‐von‐Mises test revealed violations of normality for the EPN in both experimental blocks. An aligned‐rank transform (ART)–based robust ANOVA, implemented following the Puri and Sen procedure, revealed significant main effects for both erotic and mutilation picture categories, χ^2^(16) ≥ 15.52, *p* < 0.001. Exploratory analyses using the Wilcoxon signed‐rank test showed significantly larger EPN amplitudes for high‐ compared to low‐arousing images in both the erotica and mutilation blocks across both sessions, *Z* < −3.4, *p* < 0.001.

### 2.6 Single Subject Bootstrap Analysis

EPN, LPP, and P3 data from each individual case were submitted to a bootstrap analysis (Di Nocera and Ferlazzo 2000; Efron and Tibshirani 1994; Rosenfeld 2020). Specifically, with 50,000 bootstrap repetitions, each case's EPN, LPP, and P3 mean data were resampled by randomly (re‐)assigning a case's trials to the categories of interest (i.e., high vs. low arousing for emotion effects and target vs. non‐target for the task effect) with replacement. The mean difference between the resulting ERPs was calculated for each bootstrap run. Significance (*p* < 0.05, one‐sided) on the individual case level was determined as the proportion of results in the empirical probability distribution that were equal or more extreme than the observed mean EPN, LPP, or P3 difference, respectively. *p*‐values of *p* ≤ 0.05, *p* ≤ 0.01, *p* ≤ 0.001, and *p* < 0.00002 indicate that less than or equal to 2500, 500, 50, and 0 out of 50,000 randomized calculations yielded an equal or more extreme result. To relate the bootstrapped *p*‐values to the variability of the bootstrap distribution and to allow comparison across sessions, the observed difference score of each case was *z*‐transformed as a normalized location parameter, using the formula $z=\left(\bar{x}-\mu \right)/\sigma$, where $\bar{x}$ is the observed ERP difference, μ is the mean, and *σ* is the standard deviation of the bootstrap distribution of differences (see Efron and Tibshirani 1994).

For single case analysis, the time window for each of the ERP components was allowed to vary between participants and emotion categories to acknowledge inter‐individual variability in functional brain organization. Time restrictions were a priori defined based on previous research for the emotion and target effects. Specifically, it was defined that an effect for the EPN should appear between 150 and 350 ms and for the LPP and P3 components between 350 and 750 ms. Within these temporal restrictions, a custom software determined for each individual case the time window showing the maximal average difference (EPN: negative, spanning 60 ms; LPP/P3: positive, spanning 100 ms) for high‐minus‐low arousing pictures (EPN/LPP) or targets minus non‐targets (P3).

Noteworthy, the time windows of the three ERP components were identified for each case based on an analysis with combined data from Session 1 and 2. Thus, while latency was allowed to vary between cases, each individual case was assessed with the same time windows in the analysis of Session 1 and 2. Further analyses, in which the time window of the ERP components was determined separately for each session, produced results that were highly similar to those reported in the manuscript.

Additional analyses were conducted to explore the effect of trial number on the reliability of the results. Specifically, reliability was assessed using subsets of 100, 200, and 300 trials.

## 3 Results

### 3.1 Case‐By‐Case Analysis

**FIGURE 1:**
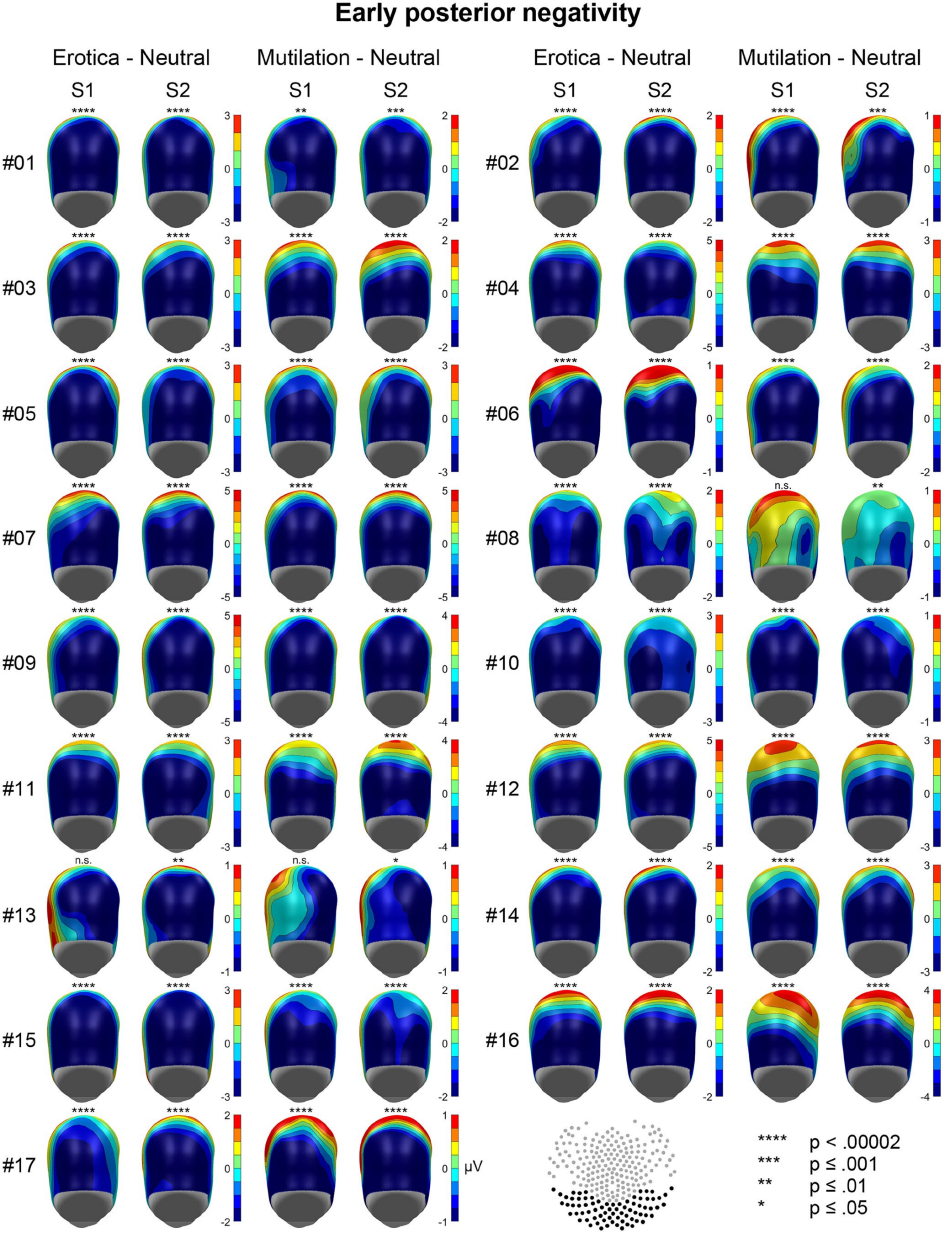
Emotional modulation of the EPN component for each individual case for the two emotion categories in each session. Map scales are individually adjusted for each participant and behavior system based on the session showing the larger effect (rounded to the nearest integer).

**FIGURE 2:**
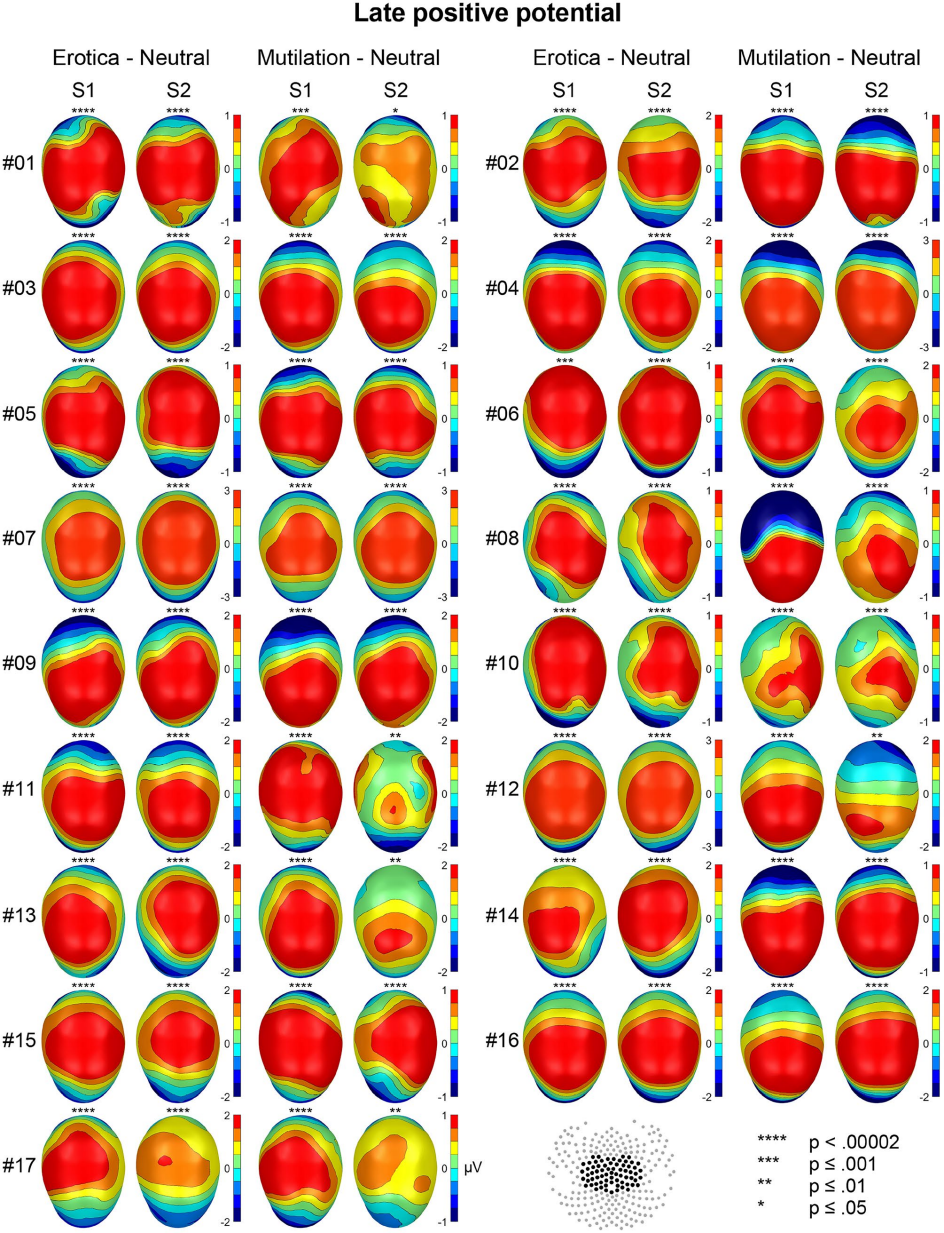
Emotional modulation of the LPP component for each individual case for the two emotion categories in each session. Map scales are individually adjusted for each participant and behavior system, based on the session showing the larger effect (rounded to the nearest integer).

**FIGURE 3:**
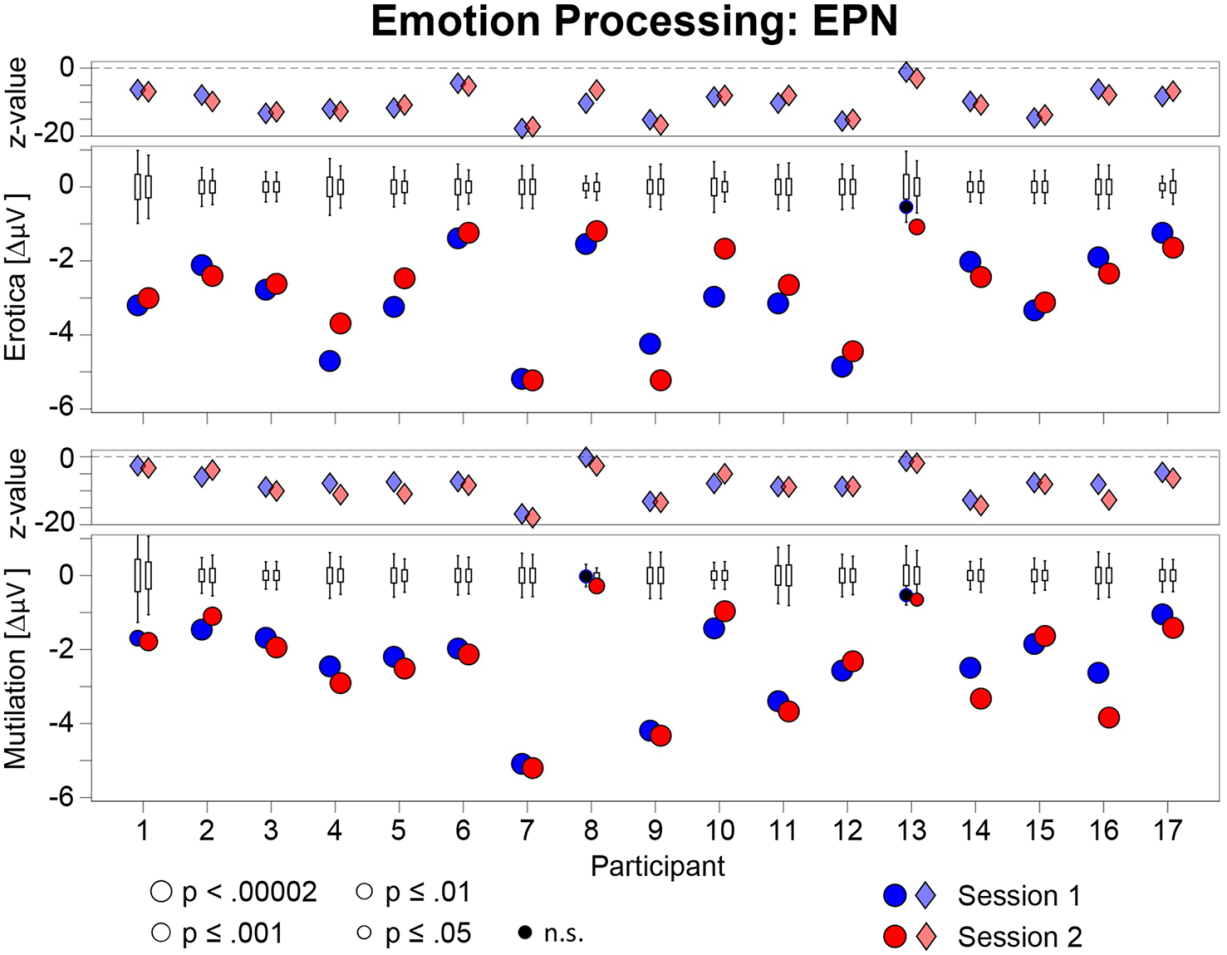
Illustration of the outcome of the case‐by‐case statistics for the emotional modulation of the EPN in the erotica and mutilation blocks for each session. Lower panels: The boxplots display the bootstrap distributions, with the bottom and top edges of each box indicating the 25th and 75th percentiles, and the whiskers indicating the 5th and 95th percentiles for each individual case. The dots indicate the measured amplitude difference (high–low arousal). Dots outside the range indicated by the whiskers represent significant effects (*p* < 0.05), with size and color indicating different p levels. Upper panels: The diamond shapes illustrate the z‐scores of individual cases for each session.

**FIGURE 4:**
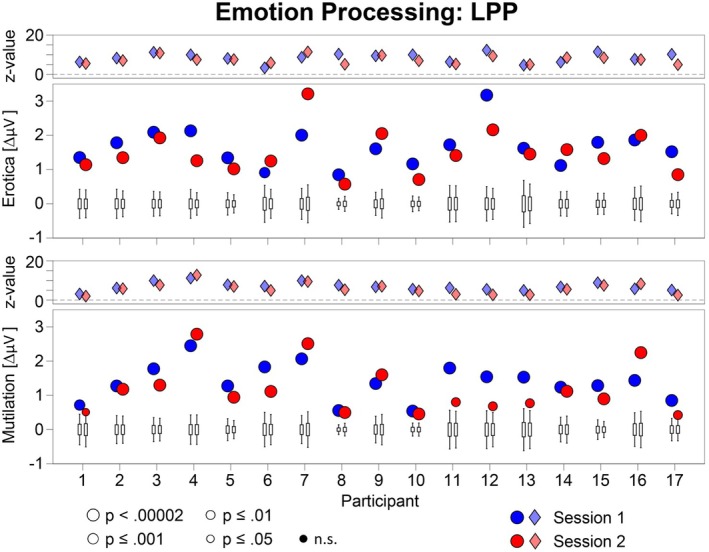
Illustration of the outcome of the case‐by‐case statistics for the emotional modulation of the LPP in the erotica and mutilation blocks for each session. Lower panels: The boxplots display the bootstrap distributions, with the bottom and top edges of each box indicating the 25th and 75th percentiles, and the whiskers indicating the 5th and 95th percentiles for each individual case. The dots indicate the measured amplitude difference (high–low arousal). Dots outside the range indicated by the whiskers represent significant effects (*p* < 0.05), with size and color indicating different p levels. Upper panels: The diamond shapes illustrate the z‐scores of individual cases for each session.

**FIGURE 5:**
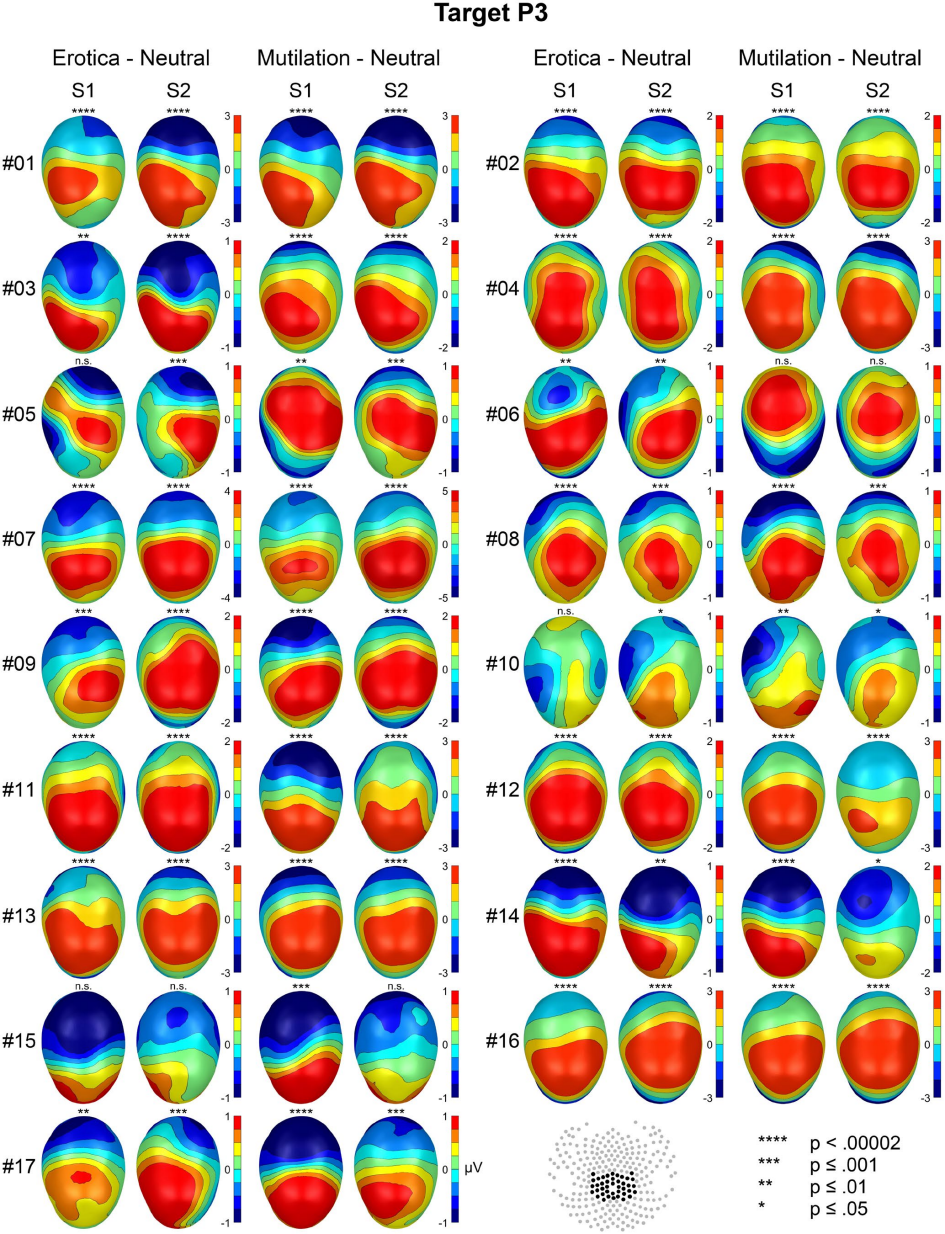
Target P3 effect for each individual case for the two emotion categories in each session. Map scales are individually adjusted for each participant and behavior system based on the session showing the larger effect (rounded to the nearest integer).

**FIGURE 6:**
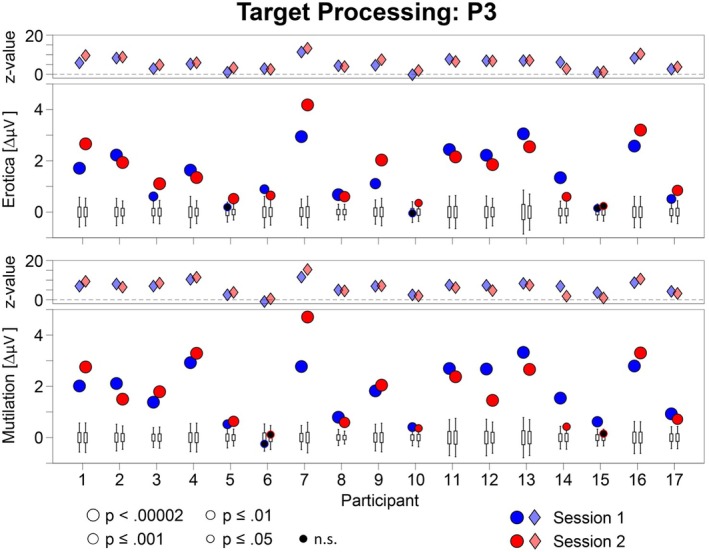
Illustration of the outcome of the case‐by‐case statistics for the target P3 effect in the erotica and mutilation blocks for each session. Lower panels: The boxplots display the bootstrap distributions, with the bottom and top edges of each box indicating the 25th and 75th percentiles, and the whiskers indicating the 5th and 95th percentiles for each individual case. The dots indicate the measured amplitude difference (target—nontarget). Dots outside the range indicated by the whiskers represent significant effects (*p* < 0.05), with size and color indicating different *p* levels. Upper panels: The diamond shapes illustrate the z‐scores of individual cases for each session.

The emotional modulation of the EPN and LPP is shown as a difference scalp map (high–low arousal) for each individual case in Figures 1 and 2, separately for behavior systems of sexual reproduction and disease avoidance. To support evaluation of temporal stability, Figures 3 and 4 present the results of the statistical analysis for single subject bootstrap statistics and *z*‐scores. Notably, the vast majority of participants showed a prototypical pattern of emotional EPN and LPP modulation at both sessions. Similar findings were observed for the target P3 effect, as illustrated in Figures 5 and 6.

#### 3.1.1 Emotion Processing: EPN

A highly consistent pattern of EPN modulation was observed for erotic picture processing, with 33 out of 34 individual tests (97%) reaching significance (Figures 1 and 3). Noteworthy, in 32 of these tests (94%), zero out of 50.000 randomized calculations of the bootstrap distribution yielded a more negative result than the measured EPN difference between erotic and neutral stimuli (*p* < 0.00002).

For mutilations, 32 out of 34 individual tests (94%) were significant, indicating a larger EPN to mutilation than neutral pictures, reaching significance at the highest level in 27 tests (79%; Figures 1 and 3). Two cases (#08 and #13) showed a variable pattern of findings reaching significance in only one of the two sessions.

#### 3.1.2 Emotion Processing: LPP

A consistent pattern of findings emerged for the LPP across cases (Figures 2 and 4). The effect of an increased LPP to erotic than neutral pictures was significant for all participants and both sessions (100%) and reached significance in 33 tests (97%) at the highest level of significance (*p* < 0.00002).

For mutilations, all participants showed a significantly larger LPP to mutilation than neutral pictures in session 1 and session 2 (100%), reaching significance in 28 tests (82%) at the highest level of significance (*p* < 0.00002; Figures 2 and 4).

#### 3.1.3 Target Processing: P3

For the erotic picture set, the target P3 effect was significant in 30 out of 34 tests (88%), reaching significance in 20 tests (59%) at the highest level of significance (*p* < 0.00002). Cases #05 and #10 showed the target effect only at one session. Case #15 showed no significant target P3 effect at both sessions (Figures 5 and 6).

For the mutilation picture set, 31 out of 34 tests revealed a significant target P3 effect (91%) overall, reaching significance in 23 tests (68%) at the highest level of significance (*p* < 0.00002). Case #15 showed the target effect only at session 1. Case #06 showed no significant target P3 effect at both sessions.

#### 3.1.4 Consistency Across Sessions

**FIGURE 7:**
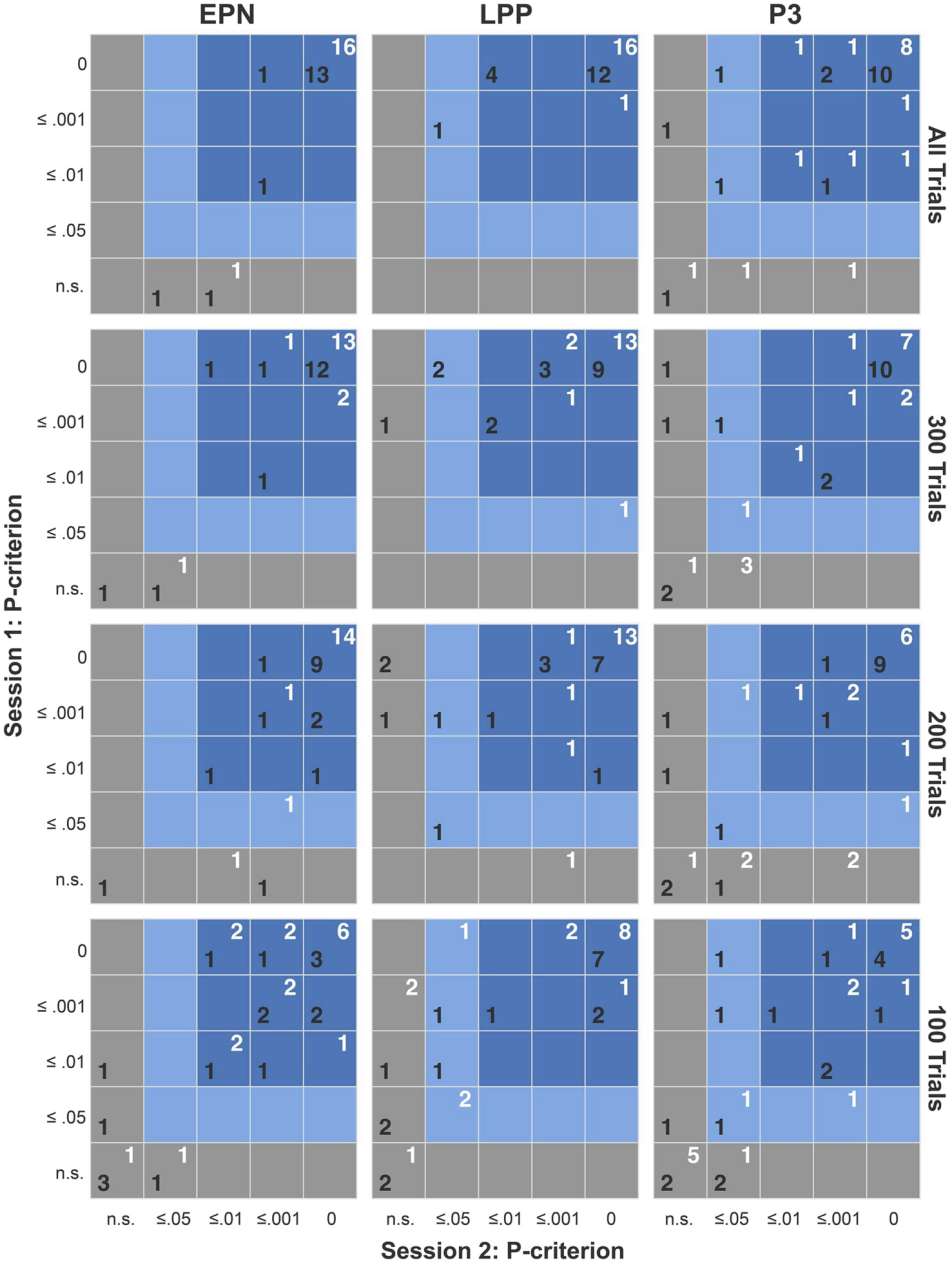
Contingency of statistical bootstrap outcomes across sessions as a function of varying P‐criteria for the EPN, LPP, and target P3 components in response to erotic (white numbers) and mutilation (black numbers) pictures. The first row displays contingencies based on all available trials per participant; subsequent rows show results when restricted to the first 300, 200, and 100 trials, respectively. Note that a criterion of *p* = 0 indicates that none of the 50,000 bootstrap samples equaled or exceeded the observed effect, corresponding to *p* < 0.00002.

Figure 7 (upper row of tables) illustrates the contingency of statistical outcomes across sessions for emotion‐ and task‐related ERP components. Specifically, 16 (94%) and 15 (88%) participants showed stable effects across sessions for the EPN in response to erotica and mutilations, that is, the effect was detected at contingent p‐criteria (*p* ≤ 0.01). Similarly, for the LPP, 17 (100%) and 16 (94%) participants consistently showed significant effects to erotica and mutilations across sessions. For the target‐P3 component, 14 (82%) and 13 (76%) participants showed contingent effects across sessions.

#### 3.1.5 Subset Analyses Based on 300, 200, and 100 Trials

While using fewer trials per condition can reduce recording time, it remains critical to determine the extent to which trial reduction is feasible without compromising signal reliability, given the square root relationship between trial count and EEG signal‐to‐noise ratio. To address this, we evaluated the effects of subsampling the full dataset to the first 300, 200, and 100 trials per condition.

As in previous research (Schupp et al. 2023), reducing the number of trials included in the analysis systematically reduced the stability of significant findings (see Figure 7). However, the extent of this reduction varied across components and conditions, reflecting differences in the statistical confidence of detecting an ERP effect under specific conditions. For example, both EPN and LPP effects—particularly in response to erotica—were consistently detected at contingent p‐criteria when analyzing 300 or 200 trials. In contrast, the target‐P3 effect demonstrated markedly lower stability. Analyses based on only 100 trials exhibited substantially reduced consistency across all components.

### 3.2 Group Analysis^2^

As shown in Figure S1, the processing of erotic and mutilation pictures was associated with enlarged EPN and LPP components, replicating previous findings. Correspondingly, target stimuli elicited an enlarged P3 component compared to nontargets. For all components, highly comparable group effects were observed across sessions.

#### 3.2.1 Emotion Processing: EPN

For the EPN, the erotic as compared to the neutral picture category was associated with a relative negative potential difference, Picture Category: (*F*(1,16) = 74.66, *p* < 0.001, $\eta_{p}^{2}$ = 0.824). Neither the main effect of Session (*F*(1,16) = 0.21, *p* = 0.653), nor the interaction of Picture Category and Session was significant (*F*(1,16) = 0.97, *p* = 0.339).

For mutilations, the main effect of Picture Category, *F*(1,16) = 47.63, *p* < 0.001, $\eta_{p}^{2}$ = 0.749, was qualified by a significant interaction with Session, *F*(1,16) = 5.11, *p* = 0.038, $\eta_{p}^{2}$ = 0.242. Post hoc tests confirmed a significantly stronger negative potential for mutilation compared to neutral images in both sessions (*p*'s < 0.001), with a slightly greater modulation in Session 2 (*t*(16) = −6.97; ΔM = −2.10) than in Session 1 (*t*(16) = −6.60; ΔM = −1.86). The main effect Session was not significant (*F*(1,16) = 0.04, *p* = 0.855).

#### 3.2.2 Emotion Processing: LPP

For the LPP, a relative positive potential difference was observed for erotic as compared to the neutral picture category (Picture Category: *F*(1,16) = 140.03, *p* < 0.001, $\eta_{p}^{2}$ = 0.897). Neither the main effect of Session (*F*(1,16) = 0.10, *p* = 0.752), nor the interaction of Picture Category by Session was significant (*F*(1,16) = 2.78, *p* = 0.115).

Processing of the mutilation as compared to the neutral images was associated with a relative positive potential difference (Picture Category: *F*(1,16) = 89.47, *p* < 0.001, $\eta_{p}^{2}$ = 0.848). Neither the main effect Session (*F*(1,16) = 0.18, *p* = 0.681), nor the interaction of Picture Category and Session was significant (*F*(1,16) = 4.37, *p* = 0.053).

#### 3.2.3 Target Processing: P3

Erotic target compared to nontarget stimuli elicited a larger P3 (Target: F(1,16) = 32.07, *p* < 0.001, $\eta_{p}^{2}$ = 0.667). Neither the main effect of Session (F(1,16) = 0.52, *p* = 0.481), nor the interaction of Session and Target was significant (F(1,16) = 0.08, *p* = 0.783).

For the mutilation picture set, target as compared to nontarget stimuli elicited a larger P3 (Target: *F*(1,16) = 29.77, *p* < 0.001, $\eta_{p}^{2}$ = 0.650), as well. Neither the main effect of Session (*F*(1,16) = 0.00, *p* = 0.983), nor the interaction of Target and Session was significant (*F*(1,16) = 0.25, *p* = 0.623).

#### 3.2.4 Test–Retest Reliability

To assess rank‐order stability of physiological measures across test–retest sessions, intra‐class correlations (ICC) were calculated using ICC(3,1), which applies a two‐way mixed‐effects model, single measures, and consistency. Findings based on ICC(2,1), which also account for mean‐level stability, were highly similar and were further complemented by additional reliability indices derived from Generalizability Theory (see Supplemental Results). Following the recommendations of Cicchetti (1994), ICC values below 0.4 are classified as poor, between 0.40 and 0.59 as fair, between 0.60 and 0.74 as good, and above 0.75 as excellent.

For the EPN, excellent test–retest reliability was observed not only for each picture category but also for the emotional difference scores (high–low arousal), reflecting robust emotional modulation effects (see Table 1).

**TABLE 1:** ICC scores and confidence intervals (CI_95_) for the EPN, LPP, and P3 component and according difference scores (high – low arousal; target – nontarget).

System	EPN	LPP		P3
Sexual reproduction				
Erotica	0.926 [0.808; 0.973]	0.558 [0.121; 0.814]	Target	0.917 [0.788; 0.969]
Neutral	0.926 [0.808; 0.973]	0.790 [0.510; 0.918]	Nontarget	0.841 [0.615; 0.939]
Δ*M* (Erotica – Neutral)	0.911 [0.773; 0.967]	0.605 [0.191; 0.836]	Δ*M* Target – Nontarget	0.873 [0.684; 0.952]
Disease avoidance				
Mutilation	0.934 [0.828; 0.976]	0.786 [0.502; 0.917]	Target	0.886 [0.714; 0.957]
Neutral	0.966 [0.908; 0.987]	0.742 [0.419; 0.898]	Nontarget	0.657 [0.272; 0.860]
Δ*M* Mutilation – Neutral	0.934 [0.827; 0.975]	0.727 [0.393; 0.892]	Δ*M* Target – Nontarget	0.865 [0.667; 0.949]

ICCs were generally lower for the LPP than for the EPN, ranging from fair to excellent across picture contents. While emotional modulation difference scores for the LPP demonstrated good temporal stability, the confidence intervals indicated substantial variability.

For the P3 component, test–retest reliability was excellent for target stimuli and good to excellent for non‐target stimuli in the erotic and mutilation picture sets, respectively. The target–non‐target difference also showed excellent test–retest reliability (see Table 1), underscoring the high temporal stability of this component at the group level.

## 4 Discussion

The present study examined the temporal stability of electrophysiological biomarkers associated with two fundamental forms of selective attention: motivated and voluntary attention. Previous research has approached this question by assessing the reliability of individual scores across repeated measurements relative to other members of a sample. Here, we adopted a different perspective, shifting from group‐level regularities and inferences about a hypothetical “average person” toward a case‐by‐case analysis. Specifically, we assessed whether an effect common to all participants can be replicated across two measurements. Overall, we found that the majority of participants showed significant emotional modulation of the EPN (88%) and LPP (100%) components, as well as a robust target P3 effect (76%) across both emotion categories and sessions. These findings suggest that neural biomarkers of selective attention can be consistently detected at the single‐case level and over a one‐week retest interval.

When analyzing a sample case‐by‐case, each individual is considered as a direct *inter*‐subject replication of the experimental effect. Furthermore, for each individual, the two picture categories yield conceptual *intra*‐subject replications testing different behavior systems (see Schupp et al. 2023; Sidman 1960). In the present study, we specifically assessed the temporal stability of ERP effects by analyzing each case of our sample at two different time points. Thus, each experimental session serves as a direct *intra*‐subject replication of the experimental effect (Sidman 1960). Using the conventional *p* ≤ 0.05 criterion, significant EPN effects were observed in 94% and 88% of cases in Session 1, and in 100% and 100% of cases in Session 2 for erotic and mutilation stimuli, respectively. LPP effects reached 100% significant cases for both behavior systems and in both sessions. Collectively, demonstrating a high level of replicability across participants and different behavior systems represents the foundation for establishing these ERP components as dependable individual‐level indicators of motivated attention.

To increase confidence in selective attention effects at the individual level, we recently proposed using experimental paradigms with multiple tasks to simultaneously assess motivated and voluntary attention (Schupp et al. 2023). Accordingly, participants performed a categorization task in which they pressed a button whenever an exemplar from the target category was presented. We detected significant target P3 effects in 82% and 94% of cases in Session 1, and in 94% and 88% of cases in Session 2, for erotic and mutilation blocks, respectively. Providing *inter*‐ and *intra*‐subject replication, these findings suggest that the target P3 effect shows a high temporal stability across participants and behavior systems.

The case‐by‐case approach also allows for inferences about individual cases. Choosing an adequate P‐criterion is of relevance for the confidence in interpretation (see Fisher 1950). At the group level, the *p*‐value is closely related to the width of the confidence interval around the point estimate and therefore provides a practical measure of confidence in rejecting the null hypothesis. By analogy, individual‐level inferences can be based on different (bootstrapped) P‐criteria, which may serve as a graded measure of confidence in detecting single‐case ERP effects under specific conditions. With an alpha level of *p* ≤ 0.01, 15 out of 17 cases (88%) showed replicable emotional EPN effects across both behavior systems and both sessions. Presumed to index the spontaneous allocation of processing resources at an early stage (Schupp et al. 2006), significant EPN effects can be interpreted with high statistical confidence as reflecting heightened motivated attention to emotionally salient stimuli. Notably, the case‐by‐case approach not only reveals empirical regularities that hold across individuals but may also capture meaningful variability at the individual level. Specifically, Case #08 exhibited a strong EPN effect in response to erotic stimuli but inconsistent findings for mutilations, indicating content‐specific differences in emotional stimulus processing. In contrast, Case #13 showed weak EPN effects that reached significance only in Session 2, suggesting a general rather than content‐specific absence of effect, which cannot be interpreted with confidence without additional data.

An equally robust pattern of findings emerged for the LPP component, thought to reflect a later processing stage associated with conscious recognition and more elaborate evaluation of significant stimuli (Schupp et al. 2006). Twelve cases showed consistent effects with *p* ≤ 0.00002. The remaining cases also exhibited consistent patterns across behavior systems and sessions (*p* < 0.05). Notably, however, these cases showed a trend toward lower LPP effects for disease avoidance in Session 2 compared to Session 1. This observation calls for further investigation with more than two repeated measurements, as such a trend could arise either randomly or from a genuine systematic decline in emotional modulation (i.e., affective habituation) within a subgroup of participants (see Ferrari et al. 2022).

With regard to voluntary attention, we found a consistent pattern of findings for the target P3 effect in response to erotic and mutilation stimuli as well as across sessions in 13 cases (*p* ≤ 0.01). Yet, two cases (#05 and #10) showed generally weak effects with inconsistent levels of significance across behavior systems and sessions, and two additional cases (#06 and #15) exhibited non‐significant effects (*p* > 0.05) across both sessions for one of the two behavior systems, respectively. These cases, arguably, offer only limited interpretative confidence and may benefit from additional repeated measurements. Overall, the consistent replication of individual‐case effects for the EPN, LPP, and P3 components supports the view that neural biomarkers of motivated and voluntary attention can be reliably detected across participants in the research sample and within individuals with high temporal stability.

The method used to score ERP components influences the interpretation of individual findings. In this study, sensor clusters were derived from group‐level analyses and applied uniformly across sessions, behavior systems, and all individuals, with identical time windows used across sessions for comparability. This conservative approach does not account for individual differences in brain structure and function (Zilles and Amunts 2010), which can alter ERP scalp topographies. Inter‐individual variability was most evident for the target P3 (Figure 5): Cases #06 and #15 were classified as stable non‐significant effects across sessions in one behavior system, yet showed atypical topographies, more anterior for #06 and more posterior for #15, rather than a true absence of the P3 effect. Individually tailored sensor clusters for the P3 might yield classifications closer to visual impressions but risk “approximate replication,” i.e., accepting findings that may not reflect the same biological effect (Kapur et al. 2012). Because our research program targets empirical regularities common to all participants, a conservative scoring strategy is most appropriate. Future work could integrate EEG with functional imaging to better characterize inter‐individual variability.

As research moves from basic studies to translational applications, methodological issues are of relevance. In a previous report (Schupp et al. 2025), we showed that a single image per behavior system may suffice for reliable detection of effects, potentially enabling individualized stimulus materials. Because statistical confidence in effect detection depends on the ratio of effect magnitude to signal quality, the present study examined the effects of reduced trial number on individual ERP replicability (see Figure 7). Some effects, such as EPN and LPP responses in the sexual reproduction system, were detectable across sessions with as few as 200–300 trials, whereas the target P3 required many more, showing declining significance already at 300 trials. Thus, if relatively large trial numbers are necessary to ensure reliability, the duration of data collection may constrain the feasibility of single‐case approaches in certain research domains. Rapid serial visual presentation (RSVP) could help address this challenge by enabling continuous image presentation and thereby achieving high trial counts within relatively short recording times (Junghöfer et al. 2001; Schupp et al. 2006). Ultimately, the efficiency of case‐by‐case approaches in translational contexts hinges on paradigm development.

## 5 Conclusion

Affective neuroscience aims to elucidate the neural mechanisms of stimulus evaluation, with progress relying on the identification of empirical regularities that link neural biomarkers to selective attention. The present study provides strong evidence that core ERP effects reflecting two fundamental forms of attentional engagement—emotional modulation of stimulus processing (EPN, LPP) and goal‐directed target effects (P3)—can be reliably detected at the single‐case level across repeated measurements. The case‐by‐case approach thus complements group‐level analyses and offers a framework for identifying stable biomarkers in healthy individuals, with potential relevance for translational affective research in developmental and clinical contexts.

## Author Contributions

**Harald. T. Schupp:** conceptualization, formal analysis, funding acquisition, investigation, methodology, supervision, software, visualization, writing – original draft, writing – review and editing. **Karl‐Philipp Flösch:** formal analysis, methodology, writing – original draft, writing – review and editing, visualization. **Ursula Kirmse:** formal analysis, investigation, methodology, software, visualization, writing – review and editing. **Tobias Flaisch:** formal analysis, investigation, methodology, software, visualization, writing – review and editing.

## Funding

This work was supported by Deutsche Forschungsgemeinschaft (Grant EXC2117‐422037984).

## Conflicts of Interest

The authors declare no conflicts of interest.

## Supporting information


**Figure S1:** Group average ERP waveforms and scalp maps illustrating (A) the emotional modulation of the EPN and LPP (high—low arousing images) and (B) the target P3 effect (target—nontarget) at both sessions. Waveforms show the average across the respective sensor clusters used in statistical analysis. Scalp maps show the mean across the analyzed time window. A back view of the model head is shown for the EPN, whereas top views are used to display the LPP and target P3 effects.
**Table S1:** EPN, LPP and P3 group mean amplitudes [CI_95%_] in μV.
**Table S2:** ICC (2,1) scores [CI_95%_] for the EPN, LPP and P3 components and according difference scores (high—low arousal; target—nontarget).
**Table S3:** Estimated variance components (G‐Study) and coefficients (D‐Study) for group mean ERP analyses.

## Data Availability

The data that support the findings of this study are available on request from the corresponding author. The data are not publicly available due to privacy or ethical restrictions.

## Endnotes

^1^
For the disease avoidance condition, high‐arousal images included: 3010, 3030, 3051, 3061, 3069, 3071, 3101, 3120, 3225, and 3550; low‐arousal images included: 2019, 2102, 2104, 2190, 2200, 2221, 2372, 2383, 2850, and 5410. For the sexual reproduction condition, high‐arousal images included: 4658, 4659, 4680, 4693, and 4694; low‐arousal stimuli included image 4610. Images not contained in the IAPS library are available upon request.

^2^
Descriptive statistics and grand average ERP waveforms from the group analysis are provided in the Supplemental Results.
