# Supplementary material for: Temporal Stability of Neural Markers of Motivated and Voluntary Attention: Case‐by‐Case and Group Analyses
Source: Psychophysiology. 2025 Dec 31;63(1):e70218. doi: 10.1111/psyp.70218 (PMC12754743; doi:10.1111/psyp.70218)
Supplement: Supplementary file 1 — Figure S1: Group average ERP waveforms and scalp maps illustrating (A) the emotional modulation of the EPN and LPP (high—low arousing images) and (B) the target P3 effect (target—nontarget) at both sessions. Waveforms show the average across the respective sensor clusters used in statistical analysis. Scalp maps show the mean across the analyzed time window. A back view of the model head is shown for the EPN, whereas top views are used to display the LPP and target P3 effects. Table S1: EPN, LPP and P3 group mean amplitudes [CI95%] in μV. Table S2: ICC (2,1) scores [CI95%] for the EPN, LPP and P3 components and according difference scores (high—low arousal; target—nontarget). Table S3: Estimated variance components (G‐Study) and coefficients (D‐Study) for group mean ERP analyses. [file PSYP-63-e70218-s001.pdf]

SUPPLEMENT

Temporal Stability of Neural Markers of Motivated and Voluntary Attention: Group and Case-by-Case Analyses

Harald. T. Schupp[1,2], Karl-Philipp Flösch[1,2], Ursula Kirmse[1] & Tobias Flaisch[1]

[1]Department of Psychology, University of Konstanz, 78457 Konstanz, Germany

[2] Centre for the Advanced Study of Collective Behaviour, University of Konstanz, 78457 Konstanz, Germany

Analysis of ERP group results

1. Grand average ERP

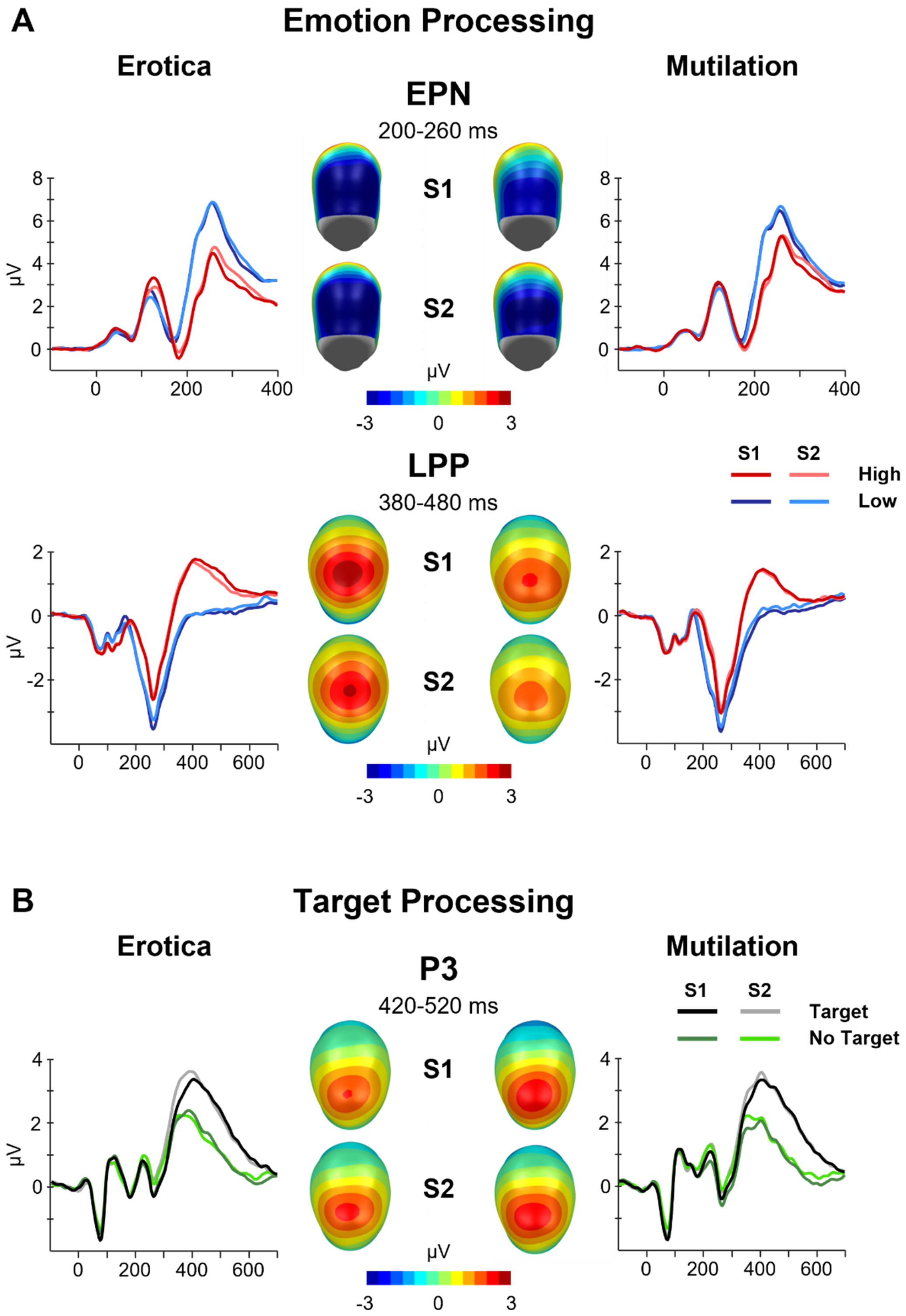

Figure S1. Group average ERP waveforms and scalp maps illustrating (A) the emotional modulation of the EPN and LPP (high – low arousing images) and (B) the target P3 effect (target – nontarget) at both sessions. Waveforms show the average across the respective sensor clusters used in statistical analysis. Scalp maps show the mean across the analyzed time window. A back view of the model head is shown for the EPN, whereas top views are used to display the LPP and target P3 effects.

Table S1: EPN, LPP and P3 group mean amplitudes [$CI_{95\%}$] in µV

EPN	Sexual Reproduction		**Erotica**	**Neutral**	**Δ*M***
		**Session 1**	2.86 [1.63; 4.09]	5.53 [4.25; 6.80]	-2.67
		**Session 2**	3.02 [1.84; 4.21]	5.56 [4.30; 6.83]	-2.54
	Disease Avoidance		**Mutilation**	**Neutral**	**Δ*M***
		**Session 1**	3.56 [2.46; 4.67]	5.42 [4.23; 6.62]	-1.86
		**Session 2**	3.41 [2.35; 4.48]	5.52 [4.32; 6.71]	-2.10
LPP	Sexual Reproduction		**Erotica**	**Neutral**	**Δ*M***
		**Session 1**	1.62 [1.22; 2.01]	0.04 [-0.26; 0.35]	1.57
		**Session 2**	1.47 [0.94; 1.99]	0.10 [-0.28; 0.47]	1.37
	Disease Avoidance		**Mutilation**	**Neutral**	**Δ*M***
		**Session 1**	1.24 [0.82; 1.65]	0.03 [-0.32; 0.37]	1.21
		**Session 2**	1.19 [0.66; 1.73]	0.18 [-0.19; 0.55]	1.01
P3	Sexual Reproduction		**Erotica**	**Neutral**	**Δ*M***
		**Session 1**	2.67 [1.89; 3.44]	1.38 [0.85; 1.92]	1.29
		**Session 2**	2.60 [1.85; 3.34]	1.27 [0.84; 1.71]	1.32
	Disease Avoidance		**Mutilation**	**Neutral**	**Δ*M***
		**Session 1**	2.74 [2.09; 3.40]	1.15 [0.84; 1.46]	1.59
		**Session 2**	2.71 [1.96; 3.46]	1.19 [0.86; 1.53]	1.51

2. Interclass-correlation results

Table S2: ICC (2,1) scores [$CI_{95\%}$] for the EPN, LPP and P3 components and according difference scores (high - low arousal; target - nontarget).

System		EPN	LPP		P3
Sexual reproduction	Erotica	.926 [.812; .972]	.558 [.132; .812]	Target	.917 [.792; .969]
	Neutral	.926 [.812; .972]	.790 [.518; .918]	Nontarget	.841 [.623; .939]
	ΔM (Erotica – Neutral)	.911 [.778; .967]	.581 [.178; .822]	ΔM (Target - Nontarget)	.873 [.691; .952]
Disease avoidance	Mutilation	.934 [.832; .975]	.786 [.511; .916]	Target	.886 [.719; .957]
	Neutral	.966 [.910; .987]	.736 [.420; .894]	Nontarget	.657 [.283; .859]
	ΔM (Mutilation – Neutral)	.919 [.758; .971]	.690 [.320; .876]	ΔM (Target - Nontarget)	.865 [.673; .948]

3. Generalizability analysis

3.1. Method

To estimate the variance components of our design and assess their respective contributions to the observed temporal stability, we conducted a generalizability study (see Cronbach, Rajaratnam, Gleser, 1963; Webb, Shavelson & Haertel, 2006; Huebner & Lucht, 2019). Specifically, we estimated a G-study with the crossed two-facet design Participant (p) × Picture Category (c) × Session (s), conducted separately for the EPN, LPP, and P3 components and for the two behavior systems of Sexual Reproduction and Disease Avoidance. As the ERP components and behavior systems represent conditions of fixed facets that cannot meaningfully be averaged across, and to maintain consistency with the group-level and single-case analyses, variance components were estimated using separate models for each component and behavior system. Accordingly, linear mixed models (LMM) were fitted using a maximum likelihood (ML) estimator based on the ERP grand mean data employed in the group analysis. All analyses were performed with R software (R Core Team 2023) using the *lmer* function from the "lme4" package (Bates et al., 2015). Each model included random intercepts for the factors *Participant*, *Picture Category* (high vs. low arousal), and *Session* (1 vs. 2), with the following specification in Wilkinson notation:

$$\text{Amplitude} \sim (1|\text{Participant}) + (1|\text{Category}) + (1|\text{Session}) + (1|\text{Participant: Category}) \\ + (1|\text{Participant: Session}) + (1|\text{Category: Session})$$

Variance components were extracted for each random effect, and their relative contributions to the total variance were expressed as percentages. Please note that the three-way interaction (Participant × Category × Session) is subsumed under the residual variance in this design. Furthermore, mean-level reliability was estimated by calculating error variances, as well as coefficients of generalizability and dependability following the procedures outlined by Webb, Shavelson, and Haertel (2006).

3.2. Results

Table S3. Estimated variance components (G-Study) and coefficients (D-Study) for group mean ERP analyses

		Sexual Reproduction						Disease Avoidance					
		EPN		LPP		P3		EPN		LPP		P3	
**Source**	**df**	σ^2	% σ^2	σ^2	% σ^2	σ^2	% σ^2	σ^2	% σ^2	σ^2	% σ^2	σ^2	% σ^2
**Participant (p)**	1,16	4.566	60.1	0.303	25.6	0.939	47.8	3.943	66.0	0.411	41.4	0.308	18.1
**Category (c)**	1,16	1.865	24.5	0.551	46.6	0.445	22.6	1.105	18.5	0.323	32.6	0.582	34.1
**Session (s)**	1,16	< 0.001	< 0.1	< 0.001	< 0.1	< 0.001	< 0.1	< 0.001	< 0.1	< 0.001	< 0.1	< 0.001	< 0.1
**Participant x Category (pc)**	1,16	0.737	9.7	0.099	8.4	0.421	21.4	0.678	11.4	0.099	10.0	0.642	37.6
**Participant x Session (ps)**	1,16	0.359	4.7	0.160	13.5	0.100	5.1	0.190	3.2	0.117	11.8	0.074	4.3
**Category x Session (cs)**	1,16	< 0.001	< 0.1	0.004	0.3	< 0.001	< 0.1	0.008	0.1	0.005	0.5	< 0.001	< 0.1
**Residual (pcs, e)**		0.071	0.9	0.065	5.5	0.061	3.1	0.048	0.8	0.037	3.7	0.100	5.9
**Error Variances** ($n'_c = 2$, $n'_s = 2$)													
**$\sigma^2(\delta)$**		0.566		0.146		0.276		0.446		0.117		0.383	
**$\sigma^2(\Delta)$**		1.500		0.422		0.498		1.000		0.280		0.674	
**Coefficients** ($n'_c = 2$, $n'_s = 2$)													
**$E\rho^2$**		0.890		0.675		0.773		0.898		0.778		0.446	
**Φ**		0.753		0.418		0.653		0.798		0.595		0.314	

Note. Relative error variance $\sigma^2(\delta) = \sigma^2_{pc}/n'_c + \sigma^2_{ps}/n'_s + \sigma^2_{pcs,e}/(n'_c\ n'_s)$. Absolute error variance $\sigma^2(\Delta) = \sigma^2_c/n'_c + \sigma^2_s/n'_s + \sigma^2_{cs}/(n'_c\ n'_s) + \sigma^2(\delta)$. The coefficient of generalizability ($E\rho^2$) was computed by $(\sigma^2_p)/(\sigma^2_p + \sigma^2(\delta))$. The coefficient of dependability (Φ) was computed by $(\sigma^2_p)/(\sigma^2_p + \sigma^2(\Delta))$.
